# Circulating microRNAs: promising candidates serving as novel biomarkers of acute hepatitis

**DOI:** 10.3389/fphys.2012.00476

**Published:** 2012-12-21

**Authors:** Natalia Elfimova, Martin Schlattjan, Jan-Peter Sowa, Hans Peter Dienes, Ali Canbay, Margarete Odenthal

**Affiliations:** ^1^Laboratory of Molecular Hepatology, Institute for Pathology, University Hospital of CologneCologne, Germany; ^2^Laboratory Ali Canbay, Department for Gastroenterology and Hepatology, University Hospital of EssenEssen, Germany

**Keywords:** microRNA, extracellular miRNA, miR-122, acute liver failure, spike-in RNA, miRNA quantification

## Abstract

Acute liver failure as life threatening condition comprises a difficult diagnostic situation to evaluate potential outcomes and therapeutic options. Thus, prognostic indicators are urgently needed for evaluation of progression of liver injury, clinical outcome, prognosis, and for therapeutic response. Recently, circulating microRNA, in particular miR-122, was described as a potential biomarker of acute liver injury after intoxication of mice. Circulating microRNA (miRNA) molecules are very stable and RNase-resistant due to protein aggregation and vesicle enclosure. Since miRNA species are known to be associated with chronic liver damage or with liver cancer, circulating miRNA patterns are suggested to serve also as reporters for progression of acute liver failure. miRNA profiling analyses using PCR arrays or next generation sequencing, may achieve identification of miRNA species that are linked to the rapid progression of acute liver injury, to the outcome of liver failure, or to the therapeutic response. Therefore, circulating miRNAs are promising, non-invasive biomarkers of future diagnostic approaches. However, normalisation of circulating miRNA levels is essential and further standardisation of miRNA quantification assays is needed.

## Introduction

Acute liver failure is a life-threatening liver disease characterized by a rapid and fulminant loss of liver function. Common causes of acute liver failure are viral hepatitis infection, mainly hepatitis A and B, and drug-induced liver intoxication (Canbay et al., 2005; Hadem et al., 2008; Ichai and Samuel, 2008, 2011; Lee, 2012). Acute liver failure demands urgent medical care, but initial symptoms of acute liver failure such as diarrhea, fatigue, and loss of appetite are rather unspecific and difficult to interpret (Renner, 2007). Rapid progression of liver malfunction is then accompanied by serious symptoms as ascites, hepatic encephalopathy, and coma. Sometimes, the acute liver failure can be reversed by valiant clinical intervention, but in many cases liver transplantation might be the only option of cure (Bernal et al., 2008; Ichai and Samuel, 2008; Lee, 2012). Thus, prognostic indicators are urgently needed for evaluation of progression of liver injury, clinical outcome, prognosis, and for therapeutic response. Recently, circulating microRNA is described as a novel tool to diagnose and monitor various diseases [summarized by Cortez and Calin (2009)].

MicroRNAs (miRNAs) are short noncoding, endogenous RNAs that regulate posttranscriptional gene expression by either RNA interference or inhibition of translational initiation and elongation (Bartel, 2004). Therefore, miRNAs are implicated in a widespread variety of cellular processes like differentiation, cell proliferation, and apoptosis (Miska, 2005; Bushati and Cohen, 2007). In human, more than thousand miRNAs are known (Kozomara and Griffiths-Jones, 2011). Mature miRNAs are formed in a step-wise process from larger primary transcripts (pri-miRNA), which are further processed, folding to hairpin structured precursor miRNA (pre-miRNA). The pre-miRNAs are exported from the nucleus, serving as substrates for the Dicer family of RNase III enzymes. One strand of the resulting short dsRNA guides the RNA-induced silencing complex (RISC) to its target mRNA (Zhao and Srivastava, 2007). Then, perfect complementary interaction of miRNAs with the untranslated region (UTR) of transcripts results in transcript degradation, whereas imperfect base pairing of miRNAs with the targeted UTR leads to translational repression (Zhao and Srivastava, 2007; Bartel, 2009).

In addition to the cellular function of miRNA in posttranscriptional gene repression, recent data collect evidence that miRNA also occur in extracellular compartments (Hunter et al., 2008; Mitchell et al., 2008). In our present review, we summarize the perspectives of circulating miRNAs to function as novel promising biomarkers of acute hepatitis.

## Dysregulation of hepatic miR-122 after liver injury

miRNA expression profiles appear to be tissue-specific. Thus, miR-122 is highly expressed in hepatocytes, due to its liver-specific transcriptional regulation by hepatocyte nuclear transcription factors (HNF1α, HNF3β, and HNF4α) (Coulouarn et al., 2009; Xu et al., 2010). miR-122, comprising approximately 70% of total miRNA in the healthy liver, takes part in liver-specific functions such as cholesterol metabolism (Esau et al., 2006; Jopling, 2012). Interestingly, miR-122 is transcribed in a circadian fashion affecting gene expression pattern of a wide range of proteins (Gatfield et al., 2009). Whereas miR-122 interaction with the 3′ untranslated region (UTR) of various transcripts leads to gene repression, miR-122 binding to two target sites in the 5′-UTR of the HCV genome results in HCV-RNA genome stabilization and enhanced replication. Hence, the liver-specific miR-122 may contribute to HCV liver tropism at the level of translation (Henke et al., 2008; Jopling, 2008).

Dedifferentiation of hepatocytes during hepatocellular carcinogenesis is associated with the loss of miR-122 (Coulouarn et al., 2009; Burchard et al., 2010; Negrini et al., 2011). In addition, during, both, acute and chronic liver damages in response to various noxa such as viral infection, drug or alcohol intoxication, or heriditary disorders, miR-122 is markedly decreased in the injured liver. Thus, after non-alcoholic fat liver diseases (Cheung et al., 2008) as well as after chronic hepatitis C infection reduced hepatic miR-122 levels were observed (Sarasin-Filipowicz et al., 2009; Morita et al., 2011). However, whereas miRNA is decreased in the injured liver, recent reports pointed to increased levels of circulating miR-122 in the blood stream after acute liver injury-induced by paracetamol intoxication of mice (Wang et al., 2009).

## miRNA released into the blood stream after liver disease

**Figure 1:**
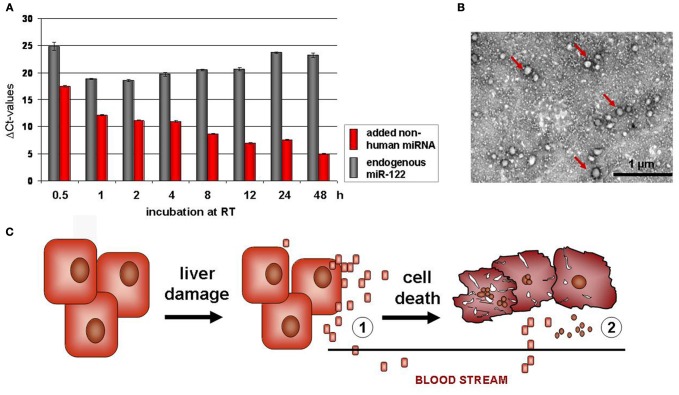
**Highly stable miRNA is released from hepatocytes after acute liver damage. (A)** High stability of endogenous miRNA in serum samples. Non-human chemically synthesized miRNA was added to human serum samples. Then the samples were incubated for 0.5 up to 48 h at room temperature (RT). Endogenous miR-122 (red) and spiked RNA (gray) was quantified by Real Time PCR, demonstrating high stability of endogenous miR-122, but degradation of the added non-human RNA. **(B)** Vesicular enclosure of circulating serum miRNA. Vesicles of around 50 nm in diameter are found in the serum, as shown by uranyl acetate negative-staining and subsequent transmission electronmicroscopy (TEM) **(B)**. The vesicle fraction carries miRNA as proven by Real Time PCR (Hunter et al., 2008; Skog et al., 2008; Chen et al., 2010). Interestingly, Bala et al. pointed out that after acute paracetamol intoxication in mice, circulating miR-122 is not predominantly associated to vesicles, but to protein aggregates (Bala et al., 2012). **(C)** Hepatic miRNA release after acute liver injury. miRNA is released from hepatocytes after acute liver damage. Interestingly, circulating miR-122 levels are increased in serum samples before levels of transaminases (ALT) were elevated. Therefore, miR-122 might not only be released after hepatocellular damage and death (2), but also by other mechanisms e.g., inflammatory processes, not yet described (1).

In serum from human patients suffering from prostate cancer, it was first described that miRNA occur also extracellularily (Hunter et al., 2008; Mitchell et al., 2008). Circulating miRNA are highly stable in serum and also RNase resistant (Figure 1A), due to protein aggregation and vesicle enclosure (Mitchell et al., 2008; Cortez and Calin, 2009; Chen et al., 2010). As vesicular structures embedding extracellular miRNA, apoptotic bodies, microvesicles (Hunter et al., 2008; Skog et al., 2008), and exosomes (Taylor and Gercel-Taylor, 2008) have been discussed (Figure 1B). Due to their high stability, they are ideal candidates considered as non-invasive diagnostic markers indicating progression and therapy outcome of disease. Accordingly, circulating miRNAs were detected in some other tumorigenic diseases such as ovarian (Lodes et al., 2009; Resnick et al., 2009), lung, and colorectal cancer (Chen et al., 2008; Ng et al., 2009) (for review please see Cortez and Calin, 2009). Furthermore, Vasilescu et al. revealed circulating miR-150 as a new prognostic marker of patients with sepsis and Wang et al. described prominent upregulation of serum miR-122 and miR-192 after acute hepatic intoxication by paracetamol in mice (Wang et al., 2009). In addition to miR-122 and miR-192, Zhou et al. identified miR-21, miR-223, miR-26a, miR-27a, and miR-801 in serum of patients with hepatocellular carcinoma (HCC) and proposed this miRNA panel as predictive markers of HCC. A comprehensive study on a wide cohort of patients with HBV or HCV based HCC revealed that high miR-25, let7f and primary miR-375 profiles only occurred in HCC-positive patients. Herein, increased miR-375 levels are shown to be specifically associated to HBV positive HCC (Li et al., 2010).

Importantly, miRNA panels in serum could not only be applied to differentiate between HCC and normal healthy donors, but also between HCC and cirrhosis (Zhou et al., 2011). Although miR-122 quantification of 68 serum samples of chronic HCV-positive patients was not normalized, the findings of Bihrer et al. definitively demonstrate that miR-122 correlated with alanine aminotransferase (ALT) values indicating liver inflammatory activity (Bihrer et al., 2011). Thus, circulating miRNAs are proposed as new biomarkers not only for tumorigenic, but also for inflammatory liver diseases.

## Extracellular miRNAs in the blood stream are promising biomarkes of acute hepatitis

Previous findings in mice after acute intoxication revealed that miR-122 increased markedly in serum samples before liver transaminases were raised (Wang et al., 2009; Zhang et al., 2010). This is of particular interest, because miRNA might not only be released by hepatocellular destruction processes, but also by active secretory delivery into the blood stream (Figure 1C). Consequently, early increased levels of serum miR-122 in response to inflammatory stimuli have to be considered as better indicators of liver failure than determination of liver-specific enzymes such as ALT (Zhang et al., 2010; Wang et al., 2012). Interestingly, Bala et al. found that after acute paracetamol intoxication of mice, miR-122 and miR-155 were predominantly associated in protein aggregates, whereas after alcoholic liver disease these two miRNAs were mainly found in the vesicular fraction (Bala et al., 2012). In addition, Novellino et al. suggested that in 13 serum samples of patients with HBV infection, miRNA is mainly complexed with the Ago2 protein, which in turn binds the hepatitis B surface protein (Novellino et al., 2012).

In addition to the experiments on acutely intoxicated mice, first data are now available on human, showing high miR-122 levels after acute hepatitis in man (Starkey Lewis et al., 2011; Ding et al., 2012). The extracellular miR-122 levels in serum from patients with acetaminophen based acute liver injury were normalized using small nuclear (sn)U6 spliceosomal RNA which is so far the most commonly applied internal reference of circulating miRNA quantification (Table 1). Though snU6 RNA is proposed to be also released into the blood stream after cellular damage including liver parenchymal injury, this data interpretation may provide a primary impression of the role of miR-122 in human acute liver failure. However, Ji et al. found no change of miR-122 in 21 patients with HBV-induced acute-on-chronic liver failure, whereas miR-122 in patients with chronic HBV infection was even decreased in comparison to 12 healthy controls (Ji et al., 2011). These conflicting results might be due to diversity of study designs and technical approaches.

**Table 1:** **miRNAs as potential biomarkers for liver disease**.

**Potential miRNA biomarker**	**Human liver disease**	**Normalisation**	**Detection/quantification method**	**Message**	**References^b^**
**High: miR-21, miR-192**, −801	HBV HCC HBV chronic (*N* ~ 1000)	miR-1228	Microarray, Real Time PCR	Differentiation between healthy donors, chronic HBV, and HCC	Zhou et al., 2011
**Low:** miR-26a, −27a, **miR-122**, −223,					
**High:** miR-25, −92a, let7f, miR-375	HBV chronic, HCC (*N* ~ 150)	/	NGS, Real Time PCR	miR-375 is HBV specific and a HCC predictor	Li et al., 2010
**High: miR-122, miR-21**, 223	HBV chronic, HCC (*N* ~ 150)	miR-181a^a^	Relative Real Time PCR	Increase in chronic HBV and HCC	Xu et al., 2011
		miR-181c^a^			
**High: miR-122**	HBV chronic (*N* = 83)	U6 RNA	Relative Real Time PCR	Increase of miR-122	Zhang et al., 2010
**High:** miR-885-5p	HBV chronic, cirrhotic, HCC (*N* > 100)	U6 RNA	Relative Real Time PCR	Increase in chronic, cirrhotic HBV and HCC	Gui et al., 2011
**High: miR-122**	HCV chronic (*N* = 68)	/	Relative Real Time PCR	Increase correlated with ALT	Bihrer et al., 2011
**High:** miR-571	Chronic HCV and alcohol (*N* = 67)	Spike-in RNA	Relative Real Time PCR	miR-571 reflects progression	Roderburg et al., 2012
**Low:** miR-652					
**High: miR-122, −34**	HCV chronic (*N* = 34), NAFLD (*N* = 35)	Spike-in RNA	Absolute Real Time PCR	Correlation with ALT, inflammatory activity and fibrosis	Cermelli et al., 2011
**High: miR-122, −192**	Acute (POD) (*N* = 53)	U6 RNA	Real Time PCR	Increase correlated with ALT	Starkey Lewis et al., 2011

a *according to geNorm; POD: acetominophen overdose*.

b *Unfortunately, we could not refer to all literature*.

*miRNA shown in bold: miRNA identified in serum samples by various reports*.

## For predictive use, standardisation in miRNA quantification is needed

**Figure 2:**
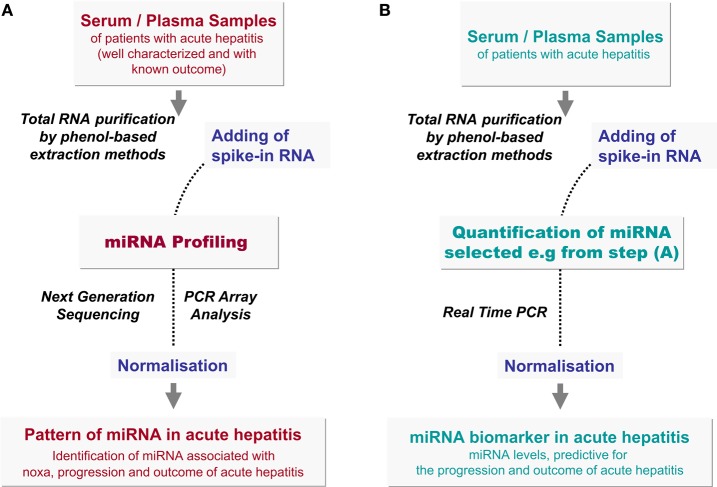
**Identification of predictive miRNAs in acute hepatitis. (A)** Retrospective studies of miRNA pattern during acute hepatitis (Discovery). For identification of putative miRNA biomarkers, well defined serum or plasma samples of patients suffering from acute hepatitis are used for total RNA isolation by means of a phenol-based extraction method. In order to normalize the levels of circulating miRNAs spike-in RNA, highly dissimilar to human miRNAs e.g., *C. elegans*, *SV-40* virus, or *Arabidopsis thaliana* or an artificial miRNA sequence, should be added to the sample before extraction. Quantitative miRNA pattern analyses can be performed by next generation sequencing (NGS) or by PCR array analyses. The correlation of miRNA profiles with clinical parameters, with disease progression and outcome will suggest a panel of miRNAs as putative indicators of hepatitis. **(B)** Analysis of selected miRNAs during acute hepatitis (Training and Validation). miRNA, identified by NGS or PCR array screening approaches, have to be validated on a wide cohort of patients with acute hepatitis by retrospective and prospective studies. For validation and future diagnostic analyses, selected miRNA are quantified by Real Time PCR (Figure 3). Normalisation of miRNA levels by spike-in RNA is essential as described in the text.

**Figure 3:**
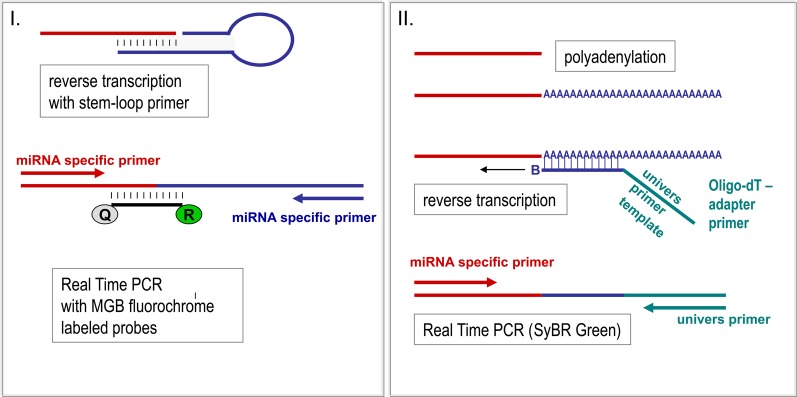
**miRNA quantification by Real Time PCR.** For PCR amplification the short miRNA molecules have to be prolonged first. Elongation of miRNA takes place simultaneously to the reverse transcription reaction by hair looped primer sets recognizing the miRNA **(I)** (Chen et al., 2005) or by unspecific polyadenylation of RNA molecules **(II)** (Shi and Chiang, 2005). Whereas in the hairpin-loop primed cDNA two specific primers are used for PCR amplification **(I)**, polyadenylated RNA, which is reversely transcribed by an oligo-dT primer carrying an universal template sequence, is amplified by the universal and only one specific primer. Real-time monitoring can be performed by integration of fluorochrome labeled probes or by interaction of fluorescent dyes with the templates. Both methods **(I** and **II)** are highly effective, though having different advantages. Whereas the usage of miRNA-specific hairpin-looped primers results in very robust and highly specific miRNA quantification, polyadenylation provides the opportunity to use cDNA from one reverse transcription reaction for analyses of several miRNAs.

Different methods are used to identify the pattern of miRNA in serum. Due to the very high sensitivity required for detection of circulating miRNA, Real Time PCR approaches are mostly carried out for screening as well as for validation of miRNA levels (Figure 2). Since miRNA are small templates, PCR needs a careful primer design and an elongation step of miRNA templates has to be combined to the reverse transcription before PCR amplification is started (Figure 3). For elongation of miRNA targets, either polyadenylation of miRNA is carried out (Shi and Chiang, 2005) or hairpin-looped primers are applied to the reverse transcription step leading to elongation of cDNA by the hairpin sequence (Chen et al., 2005) (Figure 3).

However, next generation sequencing (NGS) is also a valuable method to detect the pattern of circulating miRNAs followed by PCR quantification to validate data on a wide cohort of patients (Figure 2).

Although miR-122, miR-192, miR-21, and miR-34a are shown by most reports to be increased after experimental or human liver injury (Table 1), high variance and conflicting data exist about miRNA incidence in the blood stream upon different liver diseases. Kim et al. pointed out, that blood components that are co-purified with miRNA from serum or plasma highly affect efficiency of miRNA quantification by PCR (Kim et al., 2012). It is well-known that anti-coagulants in blood samples strongly inhibit Taq-polymerase, but plasma or serum sample volume, time until serum or plasma is prepared might also affect miRNA accessibility by Real Time PCR assays.

In addition, the accuracy of extracellular miRNA quantification highly depends on normalisation using an appropriate reference RNA. Thus, Xu et al. identified increased levels of circulating miR-122 and mir-92a as putative markers of chronic HBV infection after normalisation to endogenous miR-181 values, whereas Ji et al. observed decreasing levels of both miRNA after normalisation using snU6-RNA (Ji et al., 2011; Xu et al., 2011). snU6-RNA is mostly used as a reference. It is ubiquitously expressed in cells, but highly differs in the blood stream of different individuals (Qi et al., 2012). Furthermore, Ding et al. found that snU6-RNA is decreased after hepatocarcinogenesis (Ding et al., 2012). Hence, an internal standard for miRNA quantification is missing, so far. Therefore, the application of spike-in RNA is recommended in order to normalize errors in sample handling and extraction (Figure 2). Though miRNA are very stable molecules in serum or plasma, for better comparability and reproducibility in future studies, a well-standardized protocol is needed, in order to evaluate miRNAs as biomarkers for acute hepatitis.

### Conflict of interest statement

The authors declare that the research was conducted in the absence of any commercial or financial relationships that could be construed as a potential conflict of interest.
